# Biological rhythms are independently associated with quality of life in bipolar disorder

**DOI:** 10.1186/s40345-016-0050-8

**Published:** 2016-03-16

**Authors:** Lauren E. Cudney, Benicio N. Frey, David L. Streiner, Luciano Minuzzi, Roberto B. Sassi

**Affiliations:** Mood Disorders Program, St. Joseph’s Healthcare Hamilton, 100 West 5th St, Room C124, Hamilton, ON L8N 3K7 Canada; Women’s Health Concerns Clinic, St. Joseph’s Healthcare Hamilton, Hamilton, ON Canada; Department of Psychiatry and Behavioural Neurosciences, McMaster University, Hamilton, ON Canada; Department of Psychiatry, University of Toronto, Toronto, ON Canada

**Keywords:** Bipolar disorder, Circadian rhythm, Quality of life, Sleep

## Abstract

**Background:**

Evidence suggests that patients with bipolar disorder (BD) experience biological rhythm disturbances; however, no studies have examined the impact of this disruption on quality of life (QOL). The aim of this study is to investigate the influence of biological rhythm, depressive symptoms, sleep quality, and sleep medication use on QOL in BD.

**Methods:**

Eighty BD subjects (44 depressed and 36 euthymic) completed questionnaires assessing QOL (WHOQOL-BREF), biological rhythm disruption (BRIAN), depressive symptoms (MADRS), and sleep quality (PSQI). The impact of biological rhythm disturbance, depressive symptoms severity, sleep quality, and sleep medication use on QOL was determined with multiple regression analyses.

**Results:**

BRIAN (*β* = −0.31, *t* = −2.73, *p* < 0.01), MADRS (*β* = −0.30, *t* = −2.93, *p* < 0.01), and sleep medication use (*β* = −0.45, *t* = −2.55, *p* < 0.05) were significant predictors of QOL in this model (*F*_4, 75_ = 20.28; *p* < 0.0001). The relationship of these factors with subdomains of QOL showed that poorer social QOL was associated with greater biological rhythm disturbance (*β* = −0.43, *t* = −3.66, *p* < 0.01) and sleep medication use (*β* = −0.49, *t* = −2.35, *p* < 0.01), providing support for the social rhythm theory of BD. Physical QOL was associated with depression (*β* = −0.30, *t* = −2.93, *p* < 0.01) and biological rhythm disruption (*β* = −0.31, *t* = −2.73, *p* < 0.01). Main limitations include the cross-sectional assessment and the lack of objective measures of biological rhythms in relation to QOL.

**Conclusions:**

Disruption in biological rhythm is associated with poor QOL in BD, independent of sleep disturbance, sleep medication use, and severity of depression. Treatment strategies targeting regulation of biological rhythms, such as sleep/wake cycles, eating patterns, activities, and social rhythms, are likely to improve QOL in this population.

## Background

Bipolar disorder (BD) is a complex and debilitating disorder that affects about 4 % of the general population (Merikangas et al. 2007). BD was previously considered a cyclic disorder consisting of alterations between depressive or manic episodes and periods of euthymia. More recently, there has been a reconceptualization of BD as a chronic and multisystemic disorder, given that a substantial proportion of symptoms of BD do not completely remit even when patients are not experiencing an acute mood episode (Leboyer and Kupfer 2010). Cognitive impairment, difficulties in relationships, and social and occupational functioning also often persist during clinical remission (Giglio et al. 2010). Therefore, it is critical to understand the mechanisms that may contribute to the inter-episodic dysfunction of BD. In this regard, some of the most common problems observed during periods of remission are sleep and circadian rhythm disturbances (Harvey et al. 2005). Studies based on subjective measures of sleep quality showed that remitted BD subjects report many of the same disturbances as individuals with primary insomnia, such as decreased sleep efficiency, elevated sleep-related anxiety (Harvey et al. 2005), and more daytime sleepiness (Amand et al. 2013). A recent study showed that euthymic BD subjects experienced significantly poorer sleep quality compared to healthy controls, based on multiple components of a subjective measure of sleep quality (Rocha et al. 2013). Perhaps more importantly, sleep disturbance is the most commonly reported prodromal symptom of mania and the sixth most prominent for depression (Jackson et al. 2003).

Sleep/wake cycles follow a nearly 24-h rhythm known as circadian rhythm. Circadian rhythms can be entrained by external cues known as zeitgebers. Zeitgebers can be either physical stimuli (e.g., light) or social events such as timing of general activities or meals (Juda et al. 2013; Patton and Mistlberger 2013). Actigraphic assessments of biological rhythms in euthymic BD subjects showed greater variability in circadian activity compared to controls (Jones et al. 2005), variability of sleep duration (Millar et al. 2004), and lower daily activity levels (Salvatore et al. 2008). A recent study used actigraphy in young people with recent onset of a mood disorder, and results suggest that less robust circadian rhythmicity was associated with longer duration of illness and increasing symptom severity, suggesting this as a trait marker for young people at risk for BD (Grierson et al. 2016). Measurement of melatonin secretion, the primary hormone for circadian rhythm synchronization, also indicates changes in circadian rhythms in BD (Nathan et al. 1999). Ehlers et al. (1988) first proposed the social zeitgeber theory, in which individuals with BD are more vulnerable to social routine and sleep disturbances that may predispose them to the onset of mood episodes. Stressful life events, which disrupt routines and social rhythms (zeitgebers), can put individuals with BD at risk for a mood episode (Shen et al. 2008). Pinho et al. (2015) were the first to show that as depressive symptom severity increase, so do biological rhythm disturbance in BD patients. A recent study in a drug-naïve community sample showed that biological rhythms were more disturbed in subjects with BD compared to controls (Duarte Faria et al. 2015). Taken together, these studies show that disturbances in sleep and biological rhythms are seen throughout the course of the disorder, suggesting this disturbance can be seen as both state and trait markers of BD.

Quality of life (QOL) is a subjective measure of satisfaction with aspects of life such as physical and psychological health, social relationships, and environment (The WHOQOL Group 1995), and has consistently been shown to be decreased in BD patients (Michalak et al. 2005). Many of these QOL domains overlap with aspects that contribute to social and biological rhythms. For instance, Giglio et al. (2008) found that euthymic BD subjects with symptoms of insomnia had lower QOL than those without sleep disturbance. It has been suggested that sleep alterations may contribute to impaired QOL in BD possibly due to the effects of poor sleep quality on concentration, cognition, and memory (Giglio et al. 2008). Severity of depressive symptoms is also a strong predictor of poor QOL in BD (Amini and Sharifi 2012; Yatham et al. 2004). In fact, depressed BD patients experience more impairment in QOL than unipolar depressed patients, even when severity of depression was taken into account (Berlim et al. 2004). It has been suggested that cognitive distortions experienced during acute depression may have a negative impact on subjective ratings of QOL (Michalak et al. 2005).

As disturbances in sleep and biological rhythms are prominent features of BD, a better understanding of how these symptoms relate to overall sense of well-being and QOL is required. To the best of our knowledge, the interplay of depressive symptoms, sleep quality, and biological rhythms on QOL has not been studied in euthymic and depressed BD subjects. In addition, many psychotropic drugs used in the treatment of BD can affect sleep, but it is still unclear how these drugs may moderate the impact of sleep and biological rhythms on QOL. The objective of the present study was to examine the impact of sleep, biological rhythms, sleep medication use, and severity of depression on QOL in a well-characterized sample of euthymic and depressed BD subjects. The impact of these factors was measured on overall and specific domains of QOL such as physical, psychological, social relationships, and environment. We hypothesized that abnormalities in biological rhythms would be independently associated with poor QOL in individuals with BD.

## Methods

### Participants

Eighty patients with BD type I (*n* = 58), BD type II (*n* = 21), or NOS (*n* = 1) were recruited from the Mood Disorders Program and Women’s Health Concerns Clinic at St. Joseph’s Healthcare Hamilton, Ontario. The study was approved by the ethics committee of St. Joseph’s Healthcare Hamilton and Hamilton Health Sciences. All participants gave written informed consent to take part in the study, in accordance with declaration of Helsinki. The diagnosis of BD was confirmed with the structured clinical interview for the DSM-IV (SCID-I), as assessed by a trained research nurse and graduate student. Patients with BD were included in the study if they either met criteria for a current major depressive episode (*n* = 44) or if they did not meet criteria for any current mood episode (*n* = 36) according to the SCID-I. Patients were excluded if they met criteria for a current hypomanic, manic, or mixed episode.

### Clinical instruments

QOL was measured using the World Health Organization Quality of Life assessment (WHOQOL-BREF), which consists of 26 items, covering four domains of QOL: physical health (7 items), psychological (6 items), social relationships (3 items), and environment (8 items), with greater scores indicating better QOL (The WHOQOL Group 1995). This scale has been validated and widely used in BD research (Michalak et al. 2005). The Montgomery-Åsberg Depression Rating Scale (MADRS) was employed to measure depressive symptom severity (Montgomery and Asberg 1979). Degree of biological rhythm disruption was measured with the Biological Rhythm Interview of Assessment in Neuropsychiatry (BRIAN). The BRIAN consists of 18-items measuring sleep, overall activities, social rhythm, and eating behavior scored from 1 (no difficulties) to 4 (serious difficulties), with greater scores indicating more biological rhythm disruption. This scale has been validated in BD patients in its ability to discriminate euthymic BD and controls (Giglio et al. 2009). The BRIAN scale has previously been used in conjunction with measures of cognitive functioning in euthymic BD patients and showed that biological rhythm disruption was a predictor of poorer functioning (Giglio et al. 2010). Pittsburgh Sleep Quality Index (PSQI) was used to measure subjective sleep quality (Buysse et al. 1989). The PSQI is a standardized self-rating questionnaire which evaluates seven components of sleep quality, including subjective sleep quality, sleep latency, sleep duration, habitual sleep efficiency, sleep disturbances, use of sleeping medication, and daytime dysfunction. To more closely examine the use of sleep medications on QOL, a separate factor was included based on the answer to “use of sleep medication” (component 6). Sleep medication status was categorized as ‘yes’ if participants reported using sleep medications either ‘once or twice a week’ or ‘more than three times per week’ on component 6 of the PSQI. This response was cross-referenced with medications recorded for each participant during the clinical assessments (including bedtime use of benzodiazepines, zopiclone, quetiapine, or over-the-counter compounds such as melatonin).

### Statistical analysis

All analyses were performed with R (Version 3.1.0, R Development Core Team, 2014). Multiple regression analysis was performed using MADRS, PSQI, BRIAN, and sleep medication status as predictors, and overall WHOQOL-BREF scores as the dependent variable. To avoid colinearity between PSQI and sleep medication factor, the PSQI subscale for “use of medication” (component 6) was dropped from the total PSQI score, as previously done by (Giglio et al. 2010). Since the sample is 80 % females, sex was added to the model but was not a significant factor (*p* > 0.05). Assumptions of linear regression were tested with Shapiro–Wilk test (normality), partial residuals plots (linearity), Durbin–Watson test (independence of errors), non-constant error variance (homoscedasticity), and variance inflation factor test (multicollinearity). Each model met the assumption of multicollinearity, meaning each of the regressors was independent from each other.

To further investigate the relationships involved in the overall QOL, multiple linear regressions for each separate subdomain of the WHOQOL-BREF (physical health, psychological health, social relationships, and environment QOL) were performed, including predictors that were significant with overall WHOQOL-BREF scores. In order to control for multiple comparisons, Bonferroni correction was set at *p* = 0.05/4 = 0.0125.

## Results

Demographic and clinical data are displayed in Table 1. In patients with BD, lower QOL was correlated with greater depressive symptoms (*r*_P_ = −0.60, 95 % CI [−0.72, −0.43], *p* < 0.001), greater biological rhythm disruption (*r*_P_ = −0.60, 95 % CI [−0.72, −0.44], *p* < 0.001), and poorer sleep quality (*r*_P_ = −0.55, 95 % CI [−0.68, −0.37], *p* < 0.001). Results from the regression analyses are reported in Table 2. Multiple regression analyses indicated that BRIAN, MADRS, PSQI, and sleep medication use explained 49.4 % of the variance in total WHOQOL-BREF scores (*F*_4,75_ = 20.28; *p* < 0.0001). In this model, BRIAN (*β* = −0.31, *t* = −2.73, *p* < 0.01), MADRS scores (*β* = −0.30, *t* = −2.93, *p* < 0.01), and sleep medication use (*β* = −0.45, *t* = −2.55, *p* < 0.05) were independent predictors of QOL, whereas PSQI scores were not associated with overall QOL (*p* > 0.05).

**Table 1:** Demographic and clinical characteristics of participants

Variable	Bipolar sample (*n* = 80)
Mean age, years (SD)	42.62 (12.88)
Range	20–66
Sex	
Female	64 (80.0%)
Male	16 (20.0%)
Occupation status	
Employed	38 (47.5 %)
Unemployed	38 (47.5 %)
No data	4 (5.0 %)
Education, years (SD)	15.24 (3.11)
Current psychiatric comorbidities, *n* (%)	
Alcohol/substance abuse	11 (13.8 %)
Eating disorders	2 (2.5 %)
Generalized anxiety disorder	11 (13.8 %)
Obsessive compulsive disorder	4 (5.0 %)
Panic disorder	7 (8.8 %)
Post traumatic stress disorder	10 (12.5 %)
Social phobia	12 (15.0 %)
Specific phobia	3 (3.8 %)
Psychiatric medications, *n* (%)	
Mood stabilizers	47 (58.8 %)
Anti-psychotics	40 (50.0 %)
Antidepressants	44 (55.0 %)
Anxiolytics	40 (50.0 %)
Sleep medication use	
Yes	54 (67.5 %)
No	26 (32.5 %)
Mean total WHOQOL (SD)	202.91 (61.51)
Range	75–326
Mean WHOQOL-physical QOL (SD)	51.35 (17.69)
Range	6–88
Mean WHOQOL-psychological QOL (SD)	41.11 (20.31)
Range	0–81
Mean WHOQOL-social QOL (SD)	44.74 (25.30)
Range	0–100
Mean WHOQOL-environmental QOL (SD)	65.71 (16.48)
Range	19–100
Mean MADRS (SD)	16.76 (10.55)
Range	0–38
Mean BRIAN (SD)	47.69 (10.06)
Range	29–69
Mean PSQI (without component 6) (SD)	8.35 (3.88)
Range	2–20

**Table 2:** Multiple linear regression analysis on total WHOQOL-BREF scores

	Standardized coefficient (β)	Standard error	Unstandardized coefficients (*b*)	Standard error	*t* value	Pearson’s *r*
MADRS	−0.30**	0.10	−1.73	0.59	−2.93	−0.60**
BRIAN	−0.31**	0.11	−1.87	0.69	−2.73	−0.60**
PSQI (no meds)	−0.16	0.11	−2.61	1.70	−1.54	−0.55**
Sleep meds	−0.45*	0.17	−27.47	10.76	−2.55	n/a

Adj. *r* ^2^ = 0.494, *f* = 20.28, d*f* = 4, 75, *p* < 0.0001

* Significant at *p* < 0.05

** Significant at *p* < 0.01

Next, in order to better understand if depressive symptoms, biological rhythms, and medication use were associated with physical, social, psychological, and environmental QOL, multiple linear regressions for each WHOQOL-BREF domain were performed with MADRS, BRIAN, and sleep medication status as predictors (Table 3a–d):

**Table 3:** Multiple linear regression analysis on WHOQOL-BREF (a) physical, (b) psychological, (c) social, and (d) environmental QOL subdomain

	Standardized coefficient (β)	Standard error	Unstandardized coefficients (*b*)	Standard error	*t* value	Pearson’s *r*
(a) Physical^a^						
MADRS	−0.36**	0.11	−0.60	0.19	−3.21	−0.55**
BRIAN	−0.28**	0.11	−0.50	0.19	−2.58	−0.50**
Sleep Meds	−0.33	0.20	−5.88	3.47	−1.69	n/a
(b) Psychological^b^						
MADRS	−0.66**	0.09	−1.27	0.17	−7.31	−0.76**
BRIAN	−0.16	0.09	−0.33	0.18	−1.85	−0.54**
Sleep Meds	−0.10	0.16	−2.11	3.2051	−0.66	n/a
(c) Social^c^						
MADRS	−0.07	0.12	−0.16	0.29	−0.58	−0.36**
BRIAN	−0.43**	0.12	−1.07	0.29	−3.66	−0.49**
Sleep Meds	−0.49**	0.21	−12.41	5.28	−2.35	n/a
(d) Environmental^d^						
MADRS	0.06	0.13	12.56	25.68	0.49	−0.15
BRIAN	−0.30*	0.13	−62.46	26.42	−2.36	−0.28*
Sleep Meds	−0.56*	0.23	−1154.34	475.27	−2.43	n/a

^a^Adj. *r*_2_ = 0.3563, *f* = 15.57, d*f* = 3, 76, *p* < 0.000

^b^Adj. *r*_2_ = 0.5829, *f* = 37.8, d*f* = 3, 76, *p* < 0.0001

^c^Adj. *r*_2_ = 0.2703, *f* = 10.75, d*f* = 3, 76, *p* < 0.0001

^d^Adj. *r*_2_ = 0.1171, *f* = 4.49, d*f* = 3, 76, *p* < 0.01

* Significant at *p* < 0.05

** Significant at Bonferroni-corrected *p* < 0.0125

### Physical QOL

Physical QOL was independently associated with both MADRS (*β* = −0.36, *t* = −3.21, *p* < 0.01) and BRIAN scores (*β* = −0.28, *t* = −2.58, *p* < 0.01; Table 3a).

### Psychological QOL

Psychological QOL was strongly associated with MADRS (*β* = −0.66, *t* = −7.31, *p* < 0.01), but not with BRIAN or sleep medication use (both *p* > 0.05; Table 3b).

### Social QOL

Social QOL was independently associated with BRIAN (*β* = −0.43, *t* = −3.66, *p* < 0.01) and sleep medication status (*β* = −0.49, *t* = −2.35, *p* < 0.01), but not with MADRS (*p* > 0.05; Table 3c).

### Environment QOL

Environment QOL scores were transformed (squared) to meet the assumption of normality. Environment QOL was associated with BRIAN (*β* = −0.30, *t* = −2.36, *p* < 0.05) and sleep medication use (*β* = −0.56, *t* = −2.43, *p* < 0.05; Table 3d). However, neither of these associations were considered significant after Bonferroni correction for multiple comparisons (*p* = 0.0125).

## Discussion

This is the first study to investigate the relationship between biological rhythms and QOL in individuals with BD. The novel finding of this study is that self-reported disruption in biological rhythms was strongly associated with poor overall QOL in individuals with BD, independent of severity of depressive symptoms, sleep disturbance, and use of sleep medications. Results from the overall QOL regression (Table 2) show that despite a significant correlation between total WHOQOL-BREF scores and PSQI scores, subjective sleep quality is not significantly associated with overall QOL when depression severity, rhythm disruption, and sleep medication status are taken into account. BRIAN scale contains five questions that measure sleep disturbance as it contributes to biological rhythm disruption. It is likely that these sleep questions within the BRIAN scale are sensitive enough to account for the impact of sleep quality on QOL variation in patients with BD. Previous research implicated biological rhythm disturbance in various domains of functioning that are directly or indirectly related to QOL. For instance, Giglio et al. (2010) found that overall functioning (including measures of autonomy, work, cognition, financial, and interpersonal issues) was best predicted by biological rhythms as measured by BRIAN in euthymic BD subjects. In a large multi-center study, we have recently found that biological rhythms and depressive symptom severity were independently associated with worse psychosocial functioning in BD patients (Pinho et al. 2015). Even in periods of euthymia, BD subjects tend to report a later daily first social and work contact as compared to matched controls (Jones et al. 2005). Similarly, occupational functioning was more quickly improved in BD patients after therapy focused on regulation of social rhythms (interpersonal social rhythm therapy; IPSRT) compared to a psychoeducational therapy (Frank et al. 2008). Our results suggest that therapies targeting the regulation of biological rhythms such as IPSRT, exercise, psychoeducation, light therapy, sleep hygiene, cognitive behavioral therapy for insomnia, etc., may have a direct impact in improving QOL along with symptom recovery in BD. Considering that biological rhythm disturbance predicted decreased QOL better than sleep quality alone, this suggests that other factors associated with biological rhythms like eating patterns, activities, and social rhythms also play an important role in overall QOL.

Next, we investigated how the BRIAN, MADRS, and sleep medication use impacted each subdomain of QOL. We found that worse social QOL was significantly associated with biological rhythm disturbance (BRIAN) and use of sleep medications, but not with depression severity. Social zeitgebers that contribute to maintaining regular biological rhythms include personal relationships, and social demands and tasks (Frank et al. 2000), which are all directly associated with social relationship satisfaction. Given the findings that individuals with BD experience insecure attachment and lower levels of social support (Greenberg et al. 2014), it is to be expected that there may be alterations in social zeitgebers in these patients. This result is also consistent with the social zeitgeber theory which implies that disruptions in social cues and daily social interactions are associated with worse clinical outcomes (Ehlers et al. 1988). IPSRT utilizes the social rhythm metric for sleep/wake times, mealtimes, time spent with others, and work periods to understand where and when the rhythm disturbance is occurring (Frank et al. 2000). A recent trial of IPSRT in treating young people with BD found that targeting the stabilization of social rhythms resulted in improved social functioning (Inder et al. 2014). Whether or not the benefit of IPSRT translates into better overall QOL remains to be studied. In addition, future studies that directly assess the association between biological rhythms and social factors such as attachment, intimacy, and perceived social support are required. Overall, these results provide evidence for the importance of maintaining regular daily routines, and in turn biological rhythms, for improving life satisfaction in social relationships in patients with BD.

We also found that physical QOL was independently associated with biological rhythm disruption and depressive symptom severity. BD is conceptualized as a multisystemic chronic illness associated with higher medical burden such as increased rates of cardiovascular disease, obesity, diabetes, etc. (Leboyer and Kupfer 2010). A recent study found that BD patients had poorer physical health than healthy controls, even when sleep and physical activity were similar between the groups (McGlinchey et al. 2014). Another study investigating the relationship between social and circadian rhythm regulation and physical health in healthy individuals showed that greater discrepancies between social and circadian rhythms resulted in higher cortisol levels, poorer sleep, and greater cardiovascular risk factors (Rutters et al. 2014). To our knowledge, the relationship between biological rhythms and physical health has not been studied in BD. Understanding the mechanisms and neurobiology of this relationship is an important future direction of research in this field.

In our study, use of sleep medications was associated with poor overall QOL in BD (Table 2). The impact of sleep medication on individual domains of QOL was stronger for social QOL. Previous studies found an association between sleep medication use and impaired physical, but not psychological QOL in individuals with and without insomnia (Sasai et al. 2010). However, our results do not show a relationship between sleep medication use and physical QOL in BD patients. This may be a valid comparison to our sample of BD subjects, since sleep disturbances commonly observed in periods of euthymia in BD are comparable to those seen in individuals with primary insomnia (Harvey et al. 2005). Therefore, it is possible that the use of sleep medications is associated with poor QOL in BD because more severe forms of BD have greater sleep disturbances, which would, in turn, increase the need for use of sleep aids. Side effects of sleep aids such as sedation, fatigue, and dizziness may also play a role in decreasing QOL. Notably, preclinical studies found that mood stabilizers, such as lithium and valproate, can regulate the expression of circadian rhythm genes (Johansson et al. 2011; Yin et al. 2006). This is consistent with human studies showing that these mood stabilizers reduce the melatonin light sensitivity in healthy volunteers (Hallam et al. 2005a, b). Unfortunately, the cross-sectional nature of our study does not allow us to determine the directionality of this relationship.

Finally, we replicated previous findings that the severity of depressive symptoms is an independent predictor of poor QOL (Michalak et al. 2005). Yatham et al. (2004) previously showed that QOL was inversely correlated with severity of depression in both unipolar and bipolar depression. However, it is worth mentioning that QOL is not merely an inverse measure of depression, since QOL improvements typically lag after remission of depressive symptoms (Murray and Michalak 2012). For instance, a study on the treatment of BD depression with quetiapine found that improvement in QOL was positively correlated with improvement in depressive and anxiety symptoms and sleep quality, and that this relationship increased over time (Endicott et al. 2008). We are not aware of any study reporting longitudinal investigation of the impact of circadian rhythm improvement on QOL.

Some of the main limitations of our study include the cross-sectional design and the lack of objective measures of biological rhythms in relation to QOL. Another limitation is that BD subjects experiencing manic, hypomanic, or mixed episodes were excluded. Recent studies found that reports of QOL during manic and hypomanic episodes tend to be less impaired than during depressive or mixed states (Jansen et al. 2013; Michalak et al. 2013). As disturbance of circadian rhythms have also been associated with onset of manic episodes (Jackson et al. 2003), studies on the impact of biological rhythm disruption on QOL in manic/hypomanic subjects are warranted. A further limitation of our study is that participants with comorbid psychiatric diagnoses were included in the study (see Table 1), which may influence these results. Comorbid anxiety and substance/alcohol abuse or dependence are common in BD, and these illnesses are known to influence QOL; however, the influence on circadian rhythms remains to be studied.

A future direction of the present study is to correlate the subjective measures of biological rhythm (BRIAN) with objective measures of circadian rhythm disturbance, such as actigraphy. This would help in understanding whether disrupted schedules of sleep/wake cycles, meal timing, social rhythms, etc., are captured as disruptions in circadian rhythm measures. The BRIAN has previously been associated with increased levels of oxidative stress in BD patients (Cudney et al. 2014), but has not been evaluated with other biological measures. Sleep and circadian assessment with dim light melatonin onset (DLMO) and actigraphy have recently been studied in youth with mood symptoms: Results showed that disturbances in rhythms of melatonin and sleep quality differed in early stages of mood disorder (Naismith et al. 2012). DLMO was not associated with severity of depression or sleep quality rating scales (Naismith et al. 2012). Other studies have found that components of actigraphic measurements correlated with subjective ratings of total sleep time (Gonzalez et al. 2013), sleep latency, and phase preference (Boudebesse et al. 2014). Future studies are needed to evaluate whether objective measures of circadian rhythms such as melatonin/DLMO and actigraphic measures correspond with BRIAN scores.

## Conclusions

In conclusion, we found that self-reported biological rhythms were strongly associated with QOL, independent of  severity of depressive symptoms, sleep quality, and use of sleeping aids in a sample of well-characterized euthymic and depressed BD subjects. Social QOL was particularly associated with biological rhythm disturbances, providing further evidence for social rhythm theory of BD. Our study suggests that biological rhythms may have a direct impact on QOL, which highlights the importance of interventions that target circadian rhythms in combination with pharmacotherapy in BD. Further studies investigating the neurobiology behind circadian rhythm disturbance in BD, and how pharmacological and non-pharmacological interventions interact with these systems are encouraged.

## Abbreviations

BD: bipolar disorder
BRIAN: biological rhythm interview of assessment in neuropsychiatry
MADRS: Montgomery-Åsberg depression rating scale
PSQI: Pittsburgh Sleep Quality Index
QOL: quality of life
WHOQOL-BREF: World Health Organization Quality of Life
